# Perioperative care of the newborns with CHDs in the time of COVID-19

**DOI:** 10.1017/S1047951120001845

**Published:** 2020-06-17

**Authors:** Dilek Dilli, Irfan Taşoğlu

**Affiliations:** 1 Department of Neonatology, The Ministry of Health of Turkey, Dr Sami Ulus Maternity and Children Research and Training Hospital, University of Health Sciences of Turkey, Ankara, Turkey; 2 Department of Pediatric Cardiovascular Surgery, Yüksek İhtisas Cardiovascular Hospital of Ankara City Hospital, The Ministry of Health of Turkey, University of Health Sciences of Turkey, Ankara, Turkey

**Keywords:** Coronavirus, pandemic, newborn, CHD, perioperative care

## Abstract

Coronavirus disease 2019 (COVID-19), caused by a novel betacoronavirus (SARS-CoV-2), has led to an unexpected outbreak affecting people of all ages. The first data showed that COVID-19 could cause severe pulmonary disease, cardiac injury, and death in adults, especially the elderly and those with concomitant diseases. Currently, it was demonstrated that severe COVID-19 may also develop in neonatal age, although rarely. Newborns with CHD are known to be at high risk for increased morbidity from viral lower respiratory tract infections because of underlying anatomical cardiac lesions. There are limited data on the implications of COVID-19 on patients with cardiovascular disease, especially for those with CHD. Herein, we aimed to summarise the COVID-19-specific perioperative management issues for newborns with CHD by combining available data from the perspectives of neonatology and paediatric cardiovascular surgery.

On 17 November, 2019, the first case of coronavirus (SARS-CoV-2) infection disease (COVID-19) was reported in an adult with pneumonia in Wuhan, China.^1^ On 30 January, 2020, the World Health Organisation declared the outbreak of SARS-CoV-2 as a Public Health Emergency of International Concern.^2^ Currently, it has become a worldwide pandemic, with 6,799,713 cases and 397,388 deaths globally by 8 June, 2020.

International epidemiologic studies outline that COVID-19 may cause clinical pictures ranging from asymptomatic viral shedding to severe pneumonia and death.^3,4^ According to first data, the clinical course of the disease in children and infants was milder than in adults.^5,6^

However, on 26 April, 2020, clinicians in the United Kingdom recognised increased reports of paediatric cases presented with symptoms of severe inflammatory syndrome (multisystem inflammatory syndrome in children) resembling Kawasaki disease.^7^ It was indicated that the children tested positive for SARS-CoV-2. As of 12 May, 2020, the New York State Department of Health also identified 102 patients with similar presentations, many of whom tested positive for SARS-CoV-2 infection.^8^Additional paediatric cases presenting with multisystem inflammatory syndrome in children with a laboratory-confirmed COVID-19 disease have been reported by authorities in other countries.^9^

Non-specific symptoms may be noted in the newborns; few may progress to lower respiratory infections.^10-13^ A few newborns who tested positive soon after birth had dyspnoea, fever, pneumonia, respiratory distress syndrome, and feeding intolerance, with overall mild disease and no deaths.^14,15^

However, there are rare data specifically about infants with CHD. In newborns and children, previous cardiac surgery can be related to the risk of a more severe form of the disease, being admitted to ICU and needing intubation as well as mechanical ventilation.^16^ In a recent experience from China, the authors reported that a past medical history of CHD, bronchopulmonary hypoplasia, respiratory tract abnormalities, haemoglobinopathies, severe malnutrition, and immune system deficiency were predisposing factors for severe disease of COVID-19.^17^

Potentially, children with CHD are thought to be at high risk for developing severe COVID-19. During the pandemic, babies with CHD continue to be born with an incidence of 1 in 100 live births.^18^ About 25% of CHDs are considered critical CHDs needing surgery or other procedures within the first year of life.^19^ We can defer many medical interventions and procedures during the crisis; however, newborns’ illnesses require continuing care, especially those needing surgery in a narrow window to avoid death and provide optimal outcomes.^20^

It is suggested that COVID-19 may interact with the cardiovascular system in different ways that increase mortality and morbidity in patients with underlying heart diseases.^21-27^ A few reports showed that these viruses cause direct myocardial injury mediated via angiotensin-converting enzyme-2.^24,25^ Severe hypoxia from acute respiratory damage caused by the virus may also result in oxidative stress and cardiac injury due to increased myocardial oxygen demand.^27^

The Michigan Medicine Congenital Heart Center has identified the high risk congenital cardiac conditions for COVID-19 as single ventricle patients (hypoplastic left heart syndrome, tricuspid atresia, and double inlet left ventricle), infants less than 12 months of age, pulmonary hypertension, oxygen saturation less than 85%, patients with unrepaired complex CHD, and patients with genetic disorders potentially associated with compromised immune systems (e.g., Down Syndrome, DiGeorge Syndrome).^28^

In this paper, we aimed to summarise the COVID-19-specific perioperative management issues for newborns with CHD by combining available data in this issue from the perspectives of neonatology and cardiovascular surgery from birth to early postoperative period.

## Management at birth

### Physiologic and management considerations for the patient

Although postnatal transmission to newborns is most likely, current evidence is inconclusive regarding in utero transmission.^14^ According to recent data, SARS-CoV-2 virus has not been detected in amniotic fluid, cord blood, or breast milk of pregnant women infected with SARS-CoV-2. The incidence of the disease is lower in neonates than in adults possibly related to the difficulty of vertical transmission of coronavirus, proper handling at birth, and timely isolation of the newborn following the birth.^22-24^

As COVID-19 could cause quick deterioration of lung function, for the safety of the mothers and newborns, some authors advise performing an elective caesarean delivery.^13,29,30^ There is also debate on the timing of delivery in cases of suspected or known maternal COVID-19 infection.

As of 23 March, 2020, The American College of Obstetricians and Gynecologists recommended that COVID-19 infection itself was not an indication for early delivery and caesarean section.^31^ Delayed cord clamping was also advised (in the absence of other medical indications) in the setting of appropriate preventive measures, even in infants with CHD.

Prior to the COVID-19 pandemic, experts have recommended that the fetuses with CHD who are at high risk for haemodynamic instability at birth should be delivered at a hospital serving as a CHD centre (including perinatology, neonatology, paediatric cardiology, and paediatric cardiovascular surgery) for rapid access to an interventional catheterisation and surgery.^32^ During the COVID-19 pandemic, this recommendation continues to be valid: the final choice for the timing and mode of delivery should be planned according to risks for the mother and baby, the type of CHD, and the advice of the cardiac team.

American Academy of Pediatrics^33^ published specific recommendations on the management of neonates born to COVID-infected mothers. It is accepted that most maternal to neonatal transmission occurs at birth or shortly after via droplet contamination. It may be reasonable to separate the infant from the mother if the infant will need cardiac surgery in order to try and avoid postnatal infection. To date, as SARS-CoV-2 has not been detected in breast milk, mothers with COVID-19 can express breast milk to be fed to their babies by uninfected caregivers.^2,33^

### Workflow considerations for the healthcare team

During delivery, airborne, droplet, and contact precautions should be taken due to the increased risk of maternal virus aerosols and the potential need of resuscitation. Healthcare facilities must develop pathways for transport of these babies to the post-partum wards. The babies with respiratory distress or haemodynamic instability due to CHD should be immediately transferred to the ICU under preventive measures.^32-34^

In our country, outborn newborns with CHD have been transferred to hospitals that serve as cardiac centres for CHD through “112” Emergency Health Services. During the COVID-19 pandemic, as most neonatal units of pandemic hospitals were converted to COVID centres, suspected or confirmed cases of COVID-19 have been directed to these hospitals. In non-pandemic hospitals, we have used isolation rooms of neonatal units for this purpose in emergencies.

## Perioperative management

### Preoperative management

#### Physiologic and management considerations for the patient

In the ICU, the newborn with CHD must be closely monitored with continuous invasive blood pressure, electrocardiograph, and pulse oximetry. COVID-19 status should be immediately evaluated from the maternal history, clinical manifestations, and laboratory and radiographic testing, when necessary.

The newborns affected by COVID-19 may be asymptomatic, mild, or severely affected. The clinical symptoms are non-specific and overlap with other infections, such as fever, temperature instability, respiratory symptoms, poor feeding, and abdominal distension. Although criteria for testing for COVID-19 may vary according to geographical regions, there are some guidelines provided by Centers for Disease Control and Prevention^34,35^ and World Health Organisation.^2^ Babies born to mothers with suspected or confirmed COVID-19, critically ill newborns needing ICU care with unexplained viral pneumonia or respiratory failure, and those having a history of close contact with patients with laboratory-confirmed COVID-19 within 14 days of symptom onset should be tested for COVID-19.

If possible, during this pandemic, it is advised to perform preoperative COVID-19 testing on every patient, even asymptomatic patients, as well as their parents.^36^ The best test for SARS-CoV-2 in the peri-operative setting is a polymerase chain reaction of respiratory secretions. A positive serology will demonstrate prior exposure (or maternal status for neonates) rather than active illness, so is less useful in a peri-operative setting. Elective surgeries should be delayed until COVID-19 virus detection results are negative at least twice with a minimum of 24 hours between tests, since the first test may be false negative. The incubation period for COVID-19 is ranged between 4 and 6 days and may be as long as 14 days.^4^ If the patient’s clinical condition is favourable, it is more prudent to delay the surgery until the patient’s symptoms have improved and/or testing has been repeated (often after 14 days) and is negative. For emergency procedures, COVID-19 status of the patient should be immediately evaluated.^36^

Symptomatic and supportive treatment is the mainstay of therapy including the supply of oxygen, the maintenance of water–electrolyte, and acid–base balance. The supplementation of water and electrolytes should be appropriate, to avoid aggravating the pulmonary oedema and reduced oxygenation, especially for newborns with CHD.

On 27 May, 2020, World Health Organisation recommended that the drugs including chloroquine and hydroxychloroquine (+/− azithromycin), antivirals, immunomodulators, and plasma therapy should not be administered as treatment or prophylaxis for COVID-19, outside of the context of clinical trials.^37^ However, on 4 June, 2020, two studies of drug therapy (hydroxychloroquine and angiotensin-converting enzyme inhibitors) and COVID-19 have been retracted from two different journals, because a number of the authors were not granted access to the underlying data.^38^ Since hydroxychloroquine may increase the risk of QTc prolongation, extreme care should be taken especially in patients with CHD.^36^Initial paediatric reports suggest that in the case of severe acute respiratory distress syndrome, high-dose pulmonary surfactant, inhaled nitric oxide, high-frequency oscillatory ventilation, and extracorporeal membrane oxygenation may be useful.^11,29^

#### Workflow considerations for the healthcare team

During the current COVID-19 pandemic, healthcare authorities have advised delaying many elective procedures/surgeries to reduce the burden of the hospitals in adults. The preventive measures and treatment algorithms have been updated by authorities day by day in the light of experienced data on COVID-19. There is no specific guideline for CHD babies with COVID-19 in our country. It should be emphasised that delaying elective procedures is dependent on local/regional COVID-19 infections requiring hospitalisation, which in turn impacts resources available for the inpatient care of newborns with CHD. As surgeries in newborns with critical CHD need to be done in a timely fashion, it would be unethical to withhold life-saving cardiac surgery in a neonate in order to free up an ICU bed for an older adult with COVID-19. Therefore, decision of the time of surgery should ideally be given made by an experienced cardiac team.^39^

In the neonatal period, cardiac surgeries for CHDs with minimal risk of haemodynamic instability^32,40^ such as ductal-dependent lesions, including hypoplastic left heart syndrome, critical coarctation, severe aortic stenosis, interrupted aortic arch, and pulmonary atresia, can be shortly delayed after starting prostaglandin E1 infusion. Currently, Stephens et al^41^ suggested guidance for surgical prioritisation in newborns with CHD for COVID-19 crisis management in CHD. In Table 1, we summarise recommendations on timing of surgery for common CHDs in neonatal period and early infancy during the COVID-19 pandemic.^40,41^ It should be noted that although the newborns with ductal-dependent lesions can have surgery delayed for an extended period of time after starting prostaglandin E1 infusions, the longer these neonates are waiting for surgery, the more likely they are to develop complications, particularly those with ductal-dependent systemic blood flow (e.g., hypoplastic left heart syndrome). Furthermore, as the newborns receiving a prostaglandin E1 infusion are cared for in an ICU due to the risk of apnoea and other complications, delaying these neonatal CHD surgeries during the pandemic is likely to complicate the care of the patients.

**Table 1.** *Recommendations on timing of surgery for common CHDs in neonatal period and early infancy during the COVID-19 pandemic.^40,41^

Type of CHD	Emergent (within 24–48 hours)	Urgent (within 1–2 weeks)	Elective (>2 weeks)
PGE1-dependent PBF			
PA/IVS	– If BAV not available	– If PDA stent not available	
TOF	– Severe hypoxemia (Critical TOF) *if BAV or ductal stent not available*	– Symptomatic on medical management	
PGE1-dependent SBF			
Critical AS	– Shock unable to stabilise on PGE1 *if BAV not available*	– If able to stabilise on PGE1 *if BAV not available*	
Coarctation of Aorta	– Shock unable to stabilise on PGE1 *if angioplasty not available*	– If able to stabilise on PGE1	
Interrupted aortic arch	– Shock unable to stabilise on PGE1	– If able to stabilise on PGE1	
HLHS	– Intact, restrictive atrial septum *if BAS not available*	– Case and surgeon dependent *Ideally, before complications developed*	
Mixing Lesions			
TGA	– Intact, restrictive atrial septum *if BAS not available*	– TGA with iVS	– TGA with a large VSD and/or a large PDA
Truncus arteriosus		– If stable	
TAPVR	– Obstructive type	– Non-obstructive type, *if gradient increased*	
Aortopulmonary Window		– Uncontrolled heart failure	
Others			
Large VSD Complete AVSD			– Poor growth/congestive HF not controlled with medications – Uncontrolled HF
Ebstein anomaly		– Refractory to medical management	
AV Block	– Symptomatic, unable to medically manage/externally pace		
PDA (*premature babies*)			– Large/moderate PDA (congestive HF despite medical treatment

AVSD: atrio-ventricular septal defect, AV Block: atrioventricular block, AS: aortic stenosis, BAS: balloon atrial septostomy, BAV: balloon valvuloplasty, HF: heart failure, HLHS: hypoplastic left heart syndrome, iVS: intact ventricular septum, PBF: pulmonary blood flow, PDA: patent ductus arteriosus, PGE1=prostaglandin-E1, PS: pulmonary stenosis, SBF: systemic blood flow, VSD: ventricular septal defect, TAPVR: total anomalous pulmonary venous return, TGA: transposition of great arteria, TOF: tetralogy of Fallot

* Note: Although the newborns with ductal-dependent lesions can have surgery delayed for an extended period of time after starting PGE1 infusions, the longer these neonates are waiting for surgery, the more likely they are to develop complications, particularly those with ductal-dependent systemic blood flow (e.g., HLHS)

The need for surgery for a newborn with CHD and suspicion of COVID-19 should be prioritised by an experienced neonatologist, infectious diseases specialist, paediatric cardiologist, and cardiovascular surgeon. Timing of surgery should be planned according to resource utilisation (anticipated ventilator duration, ICU stay, blood product usage),; clinical status of the patient; risk of delaying surgery; risk of exposure for the patient, family, and healthcare staff; co-morbidities; complexity of the procedure; and recommendations of professional societies.^40,41^ Priority can be given to alternative approaches that may need less resources and/or result in shorter hospitalisation, e.g., a ductal stent opposed to a surgical systemic-to-pulmonary artery shunt.^36,41^

Aerosol-generating procedures should be performed with great caution in a negative pressure environment, if possible. Symptomatic infants who require intubation and/or positive pressure support in the ICU setting should ideally be admitted to negative pressure isolation rooms or single-patient rooms with closed doors. The air exiting a negative isolation room should be filtered with a high-efficiency particulate air filter to remove viruses and bacteria.

The American Society of Echocardiography stated that imaging should be performed according to local standards for the prevention of virus spread.^42^ Tan et al^27^ released practical guidelines about potential resource allocation for congenital cardiac catheterisation. The authors recommended reducing case volumes of paediatric/congenital catheterisation laboratories during the pandemic.

The postponement of elective cases needs to be made at the individual centre level based on the number of total (paediatric and adult) COVID-19 patients requiring hospitalisation and ICU care, other resources (such as blood products availability), and preoperative testing capacity.

Currently, in our hospitals, emergency cardiac interventions/surgeries have been permitted to proceed. However, concerning preventive measures to reduce the spread of COVID-19, elective surgeries were largely postponed in “pandemic hospitals” or directed to the “non-pandemic hospitals” serving as cardiac centres for CHD. For critical cases, planning the intervention within the current cardiac centre is recommended, after having appropriate preventive measurements. This approach can reduce the risk of contamination that may occur during patient transport.

Mechanical ventilators, anaesthesia machines, monitors, transoesophageal echocardiography probes, ultrasound machines, perfusion pumps, blood gas analysers, activated-clotting time machines, and disposable supplies in the operating room should be prepared in advance. All the necessary items such as intubation items, peripheral arterial/central venous cannulation, syringes, gauze, surgical drapes, surgical instruments, sutures, cannulas for cardiopulmonary bypass, oxygenator and circuit for cardiopulmonary bypass, prosthetic grafts, and valves should be settled before the patient enters the operating room. The high-touch equipment such as anaesthesia workstation, infusion pumps, cardiopulmonary bypass machine, cell-saver device, and heat exchangers should be wrapped with plastic sheets to facilitate decontamination.^43^

### Intraoperative management

#### Physiologic and management considerations for the patient

A newborn with or suspected of having COVID-19 should be transferred to the COVID-19 operating room under preventive measures in a separate transport incubator. The equipment used for transport should be disinfected at pre- and postoperative transportation of COVID-19 patients. During transport, the use of an in-line high-efficiency particulate air filter is recommended. The transportation of a COVID-19 patient from operating room to cardiac ICU should be performed by a personnel with protective equipment (i.e., gloves, gown, and eye protection, such as safety goggles) to protect medical staff from COVID-19.^35^

Routine monitoring should be kept on in the operating room. Since the need for aerosolising procedures like intubation and transoesophageal echocardiography for surgery, all patients should be treated as confirmed COVID-19 cases when the disease is suspected or when the test result is not yet available.

Continued invasive haemodynamic monitoring is recommended to guide fluid therapy and the use of inotropic or vasoactive medications.

Protective mechanical ventilation strategy (target tidal volume 4–6 mL/kg peak inspiratory pressure ≤25 cm H_2_O, to target SaO_2_ about 88% to 95%, and pH ≥ 7.25) should be applied in suspected/confirmed COVID-19 patients. However, this approach would only apply to COVID-19 patients with significant parenchymal lung disease. Some of these ventilator strategies would be inappropriate for neonates with critical CHD, even if they had a COVID-19 infection, if significant lung disease was not present.

Since COVID-19 patients have a high incidence of acute kidney injury, peritoneal dialysis may be necessary in the postoperative period and the care team should consider the risks and benefits of peritoneal dialysis catheter placement at the time of surgery.^41,44-46^ Blood conservation strategies should be considered for COVID-19 patients because of the high risk of coagulation dysfunction. For all patients, coagulation status should be evaluated by platelet counts/function, prothrombin time, partial thromboplastin time, international normalised ratio, and thromboelastography. Antifibrinolytic medications, preoperative haemodilution, autologous platelet-rich plasma technology, mild hypothermia or normothermia during cardiopulmonary bypass, and intraoperative blood salvage should be used to reduce the risk of transfusion-related lung injury.^41,47^

Intraoperative transoesophageal echocardiography is recommended to monitor ventricular function, volume status, and valvular diseases and guide the anaesthetic management. Right heart dysfunction might develop from increased pulmonary vascular resistance owing to pulmonary oedema, left heart dysfunction, and cardiomyopathy in COVID-19.^46^ There is a concern for transoesophageal echocardiography related to potential aerosolisation with coughing and gagging. However, this risk is negligible in an intubated neonate under general anaesthesia and so intraoperative transoesophageal echocardiography is not accepted as an aerosolising procedure. Lung ultrasonography may be useful to assess the severity of lung disease.^48^

The decision on whether to employ extracorporeal membrane oxygenation support for COVID-19-positive patients can be controversial and will depend on resource availability, co-morbidities, and pre-illness clinical status. Some experts have recommended that extracorporeal membrane oxygenation may be considered in a newborn with CHD who is positive and otherwise healthy prior to COVID infection.^28,41^

#### Workflow considerations for the healthcare team

Most operating rooms are equipped with positive-pressure systems to ensure that air travels from operating rooms to adjacent areas, thus minimising inflow of air to the room. Negative pressure rooms may increase the risk for surgical site infection, and the recommendation to change them into negative pressure rooms is controversial.^49^ COVID-19 crisis led to the construction of a negative pressure operating room in some hospitals. Although a dedicated negative pressure room is ideal for management of such patients to prevent airborne virus spreading to adjacent areas, this should be reserved for those confirmed to be infected. In our country, operating rooms of older hospitals generally have positive pressure environments. However, new city hospitals have included negative pressure operating rooms.

Hospitals should consult with their biomedical engineers to see if any operating rooms can be converted to negative pressure environments with airflow changes. A warning sign for COVID-19 should be posted outside the operating room. The feasibility of the operating room setup and a work-plan schedule is an important issue for all surgeries. Distribution of the surgical devices and anaesthetic equipment should be unique for the predefined COVID-19 operating room. All non-essential surgical and anaesthetic equipment should be taken out of the operating room. Coordination of the healthcare workers, the work-plan schedule of the COVID-19 operating room, and designated personnel working at the COVID-19 operating room should be planned day by day.

The work-plan schedule of the COVID-19 operating room should include routine universal infection prevention practices, donning and doffing personal protective equipment, and decontamination after the procedures. Table 2 shows the donning and doffing procedure for personal protective equipment according to the guidelines.^35,50^

**Table 2.** Donning and doffing procedure of personal protective equipment.^35,50^

Donning procedure	Doffing procedure
Perform hand hygiene	Sterilise hands
Put on shoe covers/boots (if applicable)	Take off shoe covers/boots (if applicable), surgical gowns, outer gloves
Put on scrubs, N95 respirators, and disposable surgical caps	Disinfect hands
Surgical hand disinfection	Take off goggles or face shields (or full face respirators) and inner gloves
Wear coverall protective gowns and inner latex gloves	Disinfect hands
Wear goggles or face shields (or full face respirators)	Take off N95 respirators and single-use surgical caps
Wear disposable surgical gowns and outer latex gloves	Disinfect hands by alcohol based hand scrub

**Figure 1.**
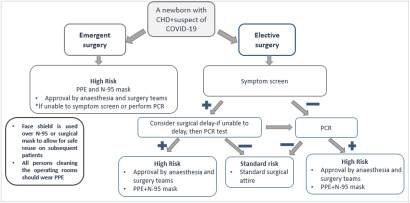
Algorithm aiming the protect surgical team members (surgeons, nurses, anaesthesiologists, technicians) in the operating room. CHD: congenital heart disease, PCR: polymerase chain reaction, PPE: personal protective equipment: gown, gloves, and eye protection

A minimum number of personnel including the anaesthesiologist, anaesthesia technician, surgeons, nurse staff, and perfusionist should be assigned before the COVID-19 patient enters the operating room. Irrelevant staff should not enter the room to minimise the traffic across the operating room. Medical personnel should enter and exit the operating room in strict accordance with the principles of clean area, contaminated pollution area, and buffer zones.^43,50^ Figure 1 shows an algorithm aiming the protect surgical team members (surgeons, nurses, anaesthesiologist, technicians) in operating room in terms of emergency of surgery and COVID status of the patient.

The staff performing tracheal intubation should be covered by personal protective equipments. Correct donning and doffing of protective equipments are essential for avoiding transmission and should be performed with a trained member of staff following local guidelines. Before intubation, avoidance of bag mask ventilation is not universally recommended as it is deemed low aerosolisation risk. Furthermore, avoidance of bag mask ventilation in neonates before laryngoscopy is very difficult in the presence of CHD. It is important that the patient coughing during laryngoscopy should be avoided with the use of adequate muscle relaxation prior to laryngoscopy. Following the intubation, ventilator is connected to the patient, before positive pressure ventilation is started. The ventilatory circuit should not be disconnected unless needed.^46^ The use of closed suction system (The Ballard® Trach Care™) designed to safely suction neonatal and children on mechanical ventilation provide protection for both the patient and the caregiver. The use of high-efficiency particulate air filters between the Ballard and the anaesthesia machine is recommended to reduce the risk of cross-infections and to prevent the contamination of the machine.^46,47^ Arterial and central venous catheterisations are recommended to be performed under ultrasound guidance to improve success rate and reduce procedure time.

For cardiac anaesthesia, sevoflurane is an agent of choice for inhalation induction. Propofol or ketamine is used for intravenous induction. The likely physiological consequences of varying systemic and pulmonary vascular resistances on shunts and cardiac output must be considered in selection of anaesthetic agents. The depth of anaesthesia should be adjusted to avoid hypotension and the need for inotropic drugs.^46,47^

After the surgery, all the surgical gowns and gloves must be taken off at the COVID-19 operating room at an allocated place for doffing. All medical waste, including breathing tubes, infusion tubing, and disposable laryngoscopes, must be sealed with double-layered medical waste bags and treated as infectious medical waste. The anaesthesia workstation should be properly sterilised according to the manufacturer recommendations.^43,46,47^

SARS-CoV-2 is known to be sensitive to ultraviolet rays and heat. Coronaviruses can be inactivated by lipid solvents, including either (75%), ethanol, chlorine-containing disinfectant, peroxyacetic acid, and chloroform except for chlorhexidine. Therefore, chlorine containing disinfectants are accepted to be appropriate to clean the operating room floor and wipe the surface of reusable medical equipments. Cleaning should be delayed until the air in the room is turned over several times according to the recommendations of biomedical engineering department based on the air handling system. Ultraviolet light decontamination for the room is recommended prior to use for a COVID-negative or unknown patient.^43,46,47^

For healthcare providers, at least 14 days of quarantine is recommended if exposure to COVID-19 occurs with inadequate personal protective equipment during care.^2,34^

### Postoperative management

#### Physiologic and management considerations for the patient

The patients should be transferred to ICU under preventive measurements as previously mentioned. Neonates undergoing cardiac surgery, with or without the use of extracorporeal circulation, as per protocol are rarely extubated in the operating room. During transport of the patients, ventilation should be performed by a disposable ambu bag or transport ventilator. The use of an in-line high-efficiency particulate air filter is recommended. The positive pressure ventilation should be stopped before disconnection from the ventilator, while placing the patient on the ambu bag. Non-invasive ventilation (e.g., Continuous Positive Airway Pressure or Bilevel Positive Airway Pressure) should be avoided as it may increase the risk of infectious transmission in patients with COVID-19 and should never be used outside of appropriate airborne/droplet isolation.^44,46,47^

##### The potential effects of COVID-19 on myocardial injury during cardiac surgery with cardiopulmonary bypass

Anaesthesia and major surgery can produce immune-inflammatory responses in patients with CHD. Systemic inflammatory response syndrome is frequently observed in children after open-heart surgery.^51^ Newborns, especially with lower birthweights, are reported to be more vulnerable to develop systemic inflammatory response syndrome following cardiopulmonary bypass. Systemic inflammatory response syndrome can impair the function of the heart, lung, kidney, liver, brain, and intestine. In a COVID-19-positive neonate, an approach that avoids the deleterious effects of cardiopulmonary bypass (i.e., pulmonary artery banding, hybrid procedure, balloon angioplasty, or valvuloplasty, etc.) may be more prudent.^36,41^

Available evidence suggests that excessive inflammation, oxidation, and an exaggerated immune response play important role in the pathogenesis of COVID-19. These events cause a cytokine storm and further progression to acute respiratory distress syndrome. The immune function of the patient has a major impact on the disease severity, and populations with low immune function, such as newborns, can be more susceptible.

**Figure 2.**
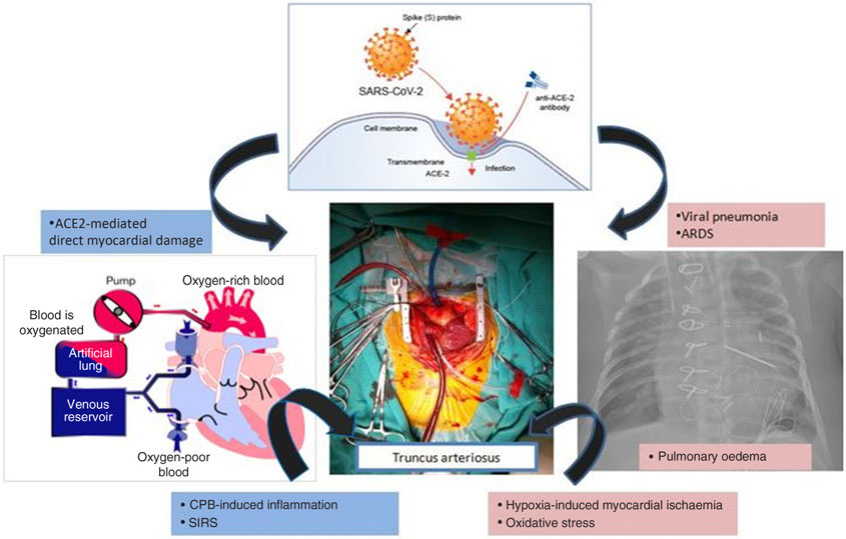
Possible mechanisms of myocardial injury in a newborn patient with COVID-19, undergoing cardiopulmonary bypass due to truncus arteriosus. CPB: cardiopulmonary bypass, ACE: angiotensin-converting enzyme, ARDS: acute respiratory distress syndrome, SIRS: systemic inflammatory response syndrome

In a recent report, it was suggested that surgery might accelerate and exacerbate the disease progression of COVID-19. Direct myocardial injury mediated via angiotensin-converting enzyme-2, viral pneumonia, acute respiratory distress syndrome, acute lung injury, hypoxia-induced myocardial damage, cardiopulmonary bypass -related systemic inflammatory response syndrome, pulmonary oedema/hypertension associated to CHD may contribute to inflammation process resulting in heart failure. Figure 2 shows possible mechanisms of myocardial injury in a newborn patient with COVID-19, undergoing cardiopulmonary bypass due to truncus arteriosus.

During cardiac surgery with cardiopulmonary bypass, accelerated inflammatory response may be reduced by off-pump cardiac surgery, control of temperature (32 to 34°C for operations <2 h of cardiopulmonary bypass), heparin coated-perfusion circuits, modified ultrafiltration, and glucocorticoids. Performing minimally invasive extracorporeal circulation and utilising the cell-saver device may both have preventive effects on the immune response, reducing the systemic cytokine load. These strategies should be kept in mind for COVID-19 patients during open cardiac surgery.

In the ICU, the COVID-19 patients should be closely monitored for systemic inflammatory response syndrome and respiratory complications and multiorgan failure. As a usual procedure, the thymus gland is routinely removed to eliminate the risk of compression on the heart and great vessels during cardiac surgery requiring a midline incision. The thymus is known to be the site of T cell development in human. However, there is no evidence that this constitutes an additional risk for infection.^28^

Anticoagulants are frequently used in cardiac surgeries. Coagulation dysfunction may develop in the course of COVID-19 infection. Therefore, modification of perioperative anticoagulation approaches (use of low-dose heparin infusions, enoxaparin use, etc.) may be considered in these patients.^44^

COVID-19 may cause acute renal injury and multiorgan failure. Since newborns also have higher levels of renal failure post-cardiopulmonary bypass, strict monitoring of fluid/nutritional intake is recommended to guide fluid therapy.^41,52^

In general, the use of corticosteroids is not recommended as it may prolong viral replication in patients with COVID-19.^36^ However, many neonates receive steroids to attenuate the inflammatory response to cardiopulmonary bypass during surgery. Therefore, steroid use should be evaluated on a patient basis considering the risk-to-benefit ratio.

In addition to supportive care, the indication of antiviral treatment should be consulted with the neonatologist and infectious diseases specialist, by the updated guideline recommendations. There may be a role for convalescent plasma (plasma from patients who have had COVID19 and now have antibodies) during neonatal critical illness.^36^

The newborn patients born to mother with COVID-19 can be fed with breastmilk (bottle or nasogastric tube) by an uninfected caregiver.^2,14,53^

During the SARS-CoV-2 pandemic, for the health and safety of the patients, parental visiting should be limited. Of course, visitor restrictions may vary at different centres depending on local and regional COVID-19 infection rates in the community. All visitors and caregivers must wear a mask at all times.^2,34,35^

##### Recommendations for frequently used drugs in COVID-19 newborn patients with CHD^21,28,36,54^

*Angiotensin-converting enzyme inhibitors*: Some infants with CHD and heart failure may be on angiotensin-converting enzyme inhibitors (e.g., captopril, enalapril, lisinopril). Angiotensin-converting enzyme inhibitors have been shown to upregulate angiotensin-converting enzyme-2 expression. Angiotensin-converting enzyme-2 counteracts the effects of angiotensin II. As SARS-CoV-2 uses angiotensin-converting enzyme-2 receptor to enter human cells, it is supposed that angiotensin-converting enzyme inhibitors may affect the severity of coronavirus infections. There is still no evidence that the use of angiotensin-converting enzyme inhibitors affects the activity of virus. The British Cardiovascular Society, British Society for Heart Failure and European Society of Cardiology Council on Hypertension, and Turkish Society of Cardiology have said that there is no clinical or scientific evidence to suggest that treatment with an angiotensin-converting enzyme inhibitor should be discontinued because of COVID-19. Stopping these medications may cause a worsening of the underlying heart condition.

*Aspirin*: It is recommended that patients who are taking aspirin should continue doing so unless advised differently by their cardiac team.

*Use of paracetamol versus ibuprofen:* Angiotensin-converting enzyme-2 expression can also be increased by ibuprofen. Nonsteroidal antinflammatory drugs are rarely if ever used in neonates and young infants. Clinicians may consider prescribing paracetamol rather than ibuprofen. This advice is offered as a precaution, although there is no significant scientific evidence that ibuprofen is associated with worse outcomes in the course of COVID-19.

*Immunisations:* It is recommended that all infants remain up to date on required immunisations.

#### Workflow considerations for the healthcare team

The number and the shift of the healthcare workers should be arranged according to the conditions of the healthcare facilities. COVID-19 newborn patients should be followed up by a dedicated cardiac COVID-19 team including an anaesthesiologist, cardiovascular surgeon, paediatric cardiologist, neonatologist, and infectious diseases specialist. The multidisciplinary approach can reduce expert bias for postoperative care. In this digital age, it may be considered as an option to perform the visits and consultations more widely by using applications such as video call telemedicine instead of in-person routine visits to prevent possible nosocomial COVID-19.

## Conclusion

In this review, we summarised the COVID-19-specific perioperative management issues for newborns with CHD by combining available data in this issue from the perspectives of neonatology and cardiovascular surgery. Prioritisation and appropriate timing of surgery are necessary at this time. Guidance strategies range from ensuring safety for specific lesions of the newborns, to maintaining availability of the staff.

## Summary

- During the current COVID-19 pandemic, the optimal timing of cardiac interventions/surgical procedures of newborns with critical CHD must be identified by the cardiac team, and options of the parents should be taken into account.
- The emergency procedures should be performed under strict preventive measures for COVID-19-positive patients.
- Special attention should be paid to COVID-19-specific cardiac and pulmonary manifestations, especially for newborns undergoing open cardiac surgery with CBP.
- In this digital age, it seems rational to perform the visits and consultations more widely by using applications such as video call telemedicine instead of in-person routine visits to prevent possible nosocomial COVID-19 infection.
- All the recommendations in this article should be reconsidered individually for each patient in the context of the dynamic changes within a given institution, population base, and geographic location.
- Circumstances are rapidly changing, even hourly, therefore, neonatologists, paediatric cardiologists, and paediatric cardiovascular surgeons should follow current online or published, national and universal data on the care of the newborns with CHD and COVID-19.
